# Supplementary material for: 3D-Printed Medical-Grade Polycaprolactone (mPCL) Scaffold for the Surgical Treatment of Vaginal Prolapse and Abdominal Hernias
Source: Bioengineering (Basel). 2023 Oct 24;10(11):1242. doi: 10.3390/bioengineering10111242 (PMC10669821; doi:10.3390/bioengineering10111242)
Supplement: Supplementary file 1 [file bioengineering-10-01242-s001.zip › bioengineering-2583934-supplementary.pdf]

Supplementary Material

Table S1. Description of mesh sizes before and after implantation measured with a ruler.

ID	Time point	Mesh location	Mesh	Mesh Size	Extraction	Notes
1787	3 months	Abdominal mesh upper	PP	30x30 mm	~28x30 mm	
	3 months	Abdominal mesh lower	mPCL	30x30 mm	~25x35 mm	
	3 months	Vaginal mesh distal	PP	20x20 mm	~20x20 mm	
	3 months	Vaginal mesh proximal	mPCL	20x20 mm	~20x15 mm	
1788	3 months	Abdominal mesh upper	PP	30x30 mm	~30x30 mm	
	3 months	Abdominal mesh lower	mPCL	30x30 mm	~30x30 mm	
	3 months	Vaginal mesh distal	PP	20x20 mm	~20x20 mm	
	3 months	Vaginal mesh proximal	mPCL	20x20 mm	~20x20 mm	
1794	3 months	Abdominal mesh upper	mPCL	30x30 mm	~35x35x20x24 mm	Not square
	3 months	Abdominal mesh lower	PP	30x30 mm	~30x30 mm	
	3 months	Vaginal mesh distal	mPCL	20x20 mm	~11x20 mm	
	3 months	Vaginal mesh proximal	PP	20x20 mm	~20x20 mm	
1795	3 months	Abdominal mesh upper	mPCL	30x30 mm	~24x26 mm	
	3 months	Abdominal mesh lower	PP	30x30 mm	~30x30 mm	
	3 months	Vaginal mesh proximal	mPCL	20x20 mm	~22x13 mm	
	3 months	Vaginal mesh distal	PP	20x20 mm	~35x5 mm	
1784	6 months	Abdominal mesh upper	mPCL	25x25 mm	NA	
	6 months	Abdominal mesh lower	PP	20x20 mm	~19x19 mm	
	6 months	Vaginal mesh proximal	pp	20x20 mm	~23x24 mm	
	6 months	Vaginal mesh distal	mPCL	20x20 mm	NA	
1783	6 months	Abdominal mesh upper	mPCL	30x30 mm	NA	
	6 months	Abdominal mesh lower	PP	30x30 mm	~30x22 mm	
	6 months	Vaginal mesh proximal	pp	20x20 mm	~19x19 mm	
	6 months	Vaginal mesh distal	mPCL	20x20 mm	~20x20 mm	

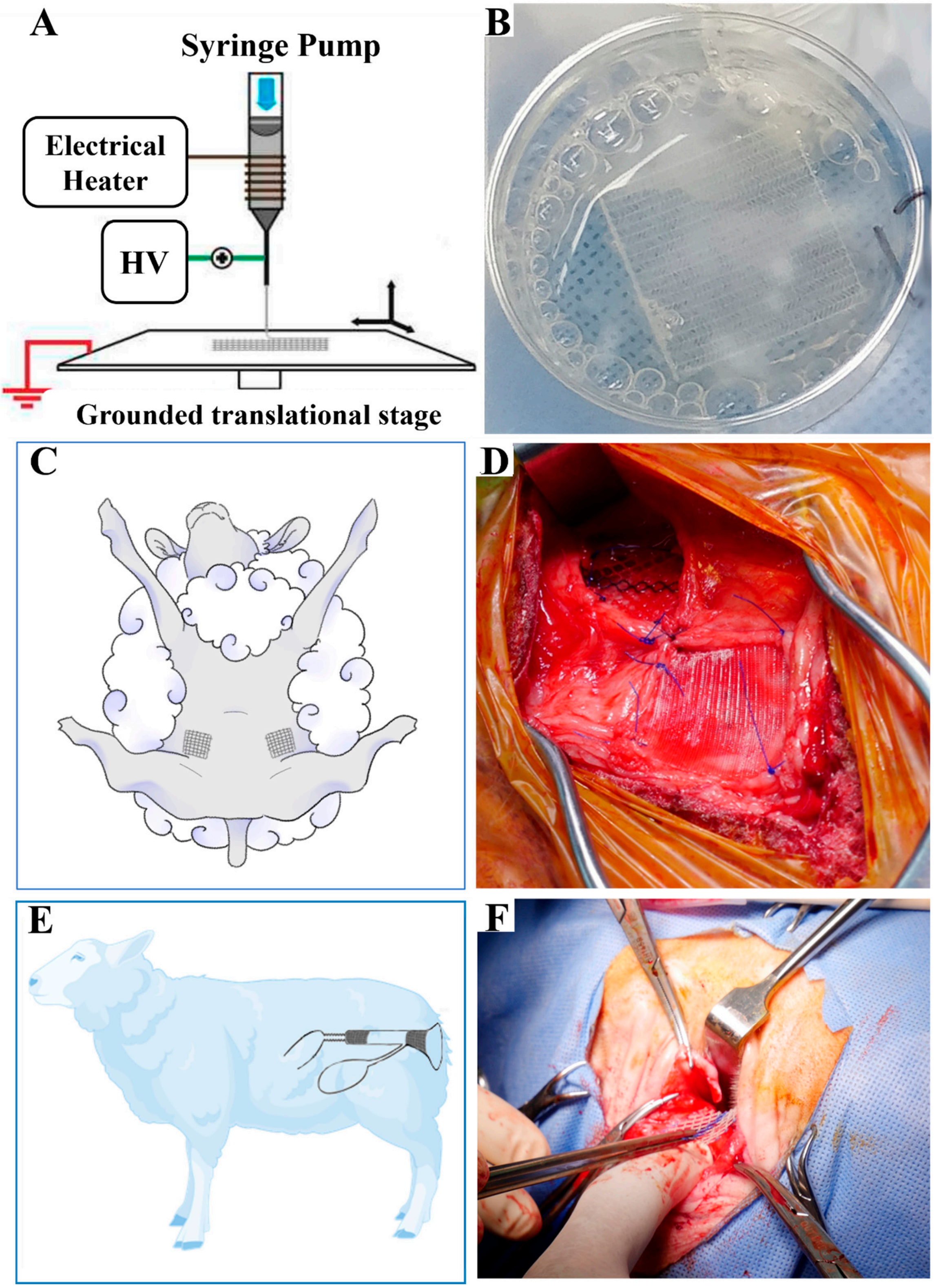

Figure S1. (**A**) Melt electrowriting (MEW) (Reproduced from Bas, O. et al.[53]), a technology combining additive manufacturing and electrospinning principles, was used to fabricate the (**B**) Medical grade polycaprolactone (mPCL) mesh scaffolds. Each sheep was implanted in their abdominal wall (**C**) with one PP mesh and one mPCL mesh scaffold (**D**); and in their vaginal floor (**D**) with one PP mesh and one mPCL mesh scaffold (**E**). Image (**D**) was partially created with BioRender.com. Anatomical structure in image (D) was modified with permission from [54].

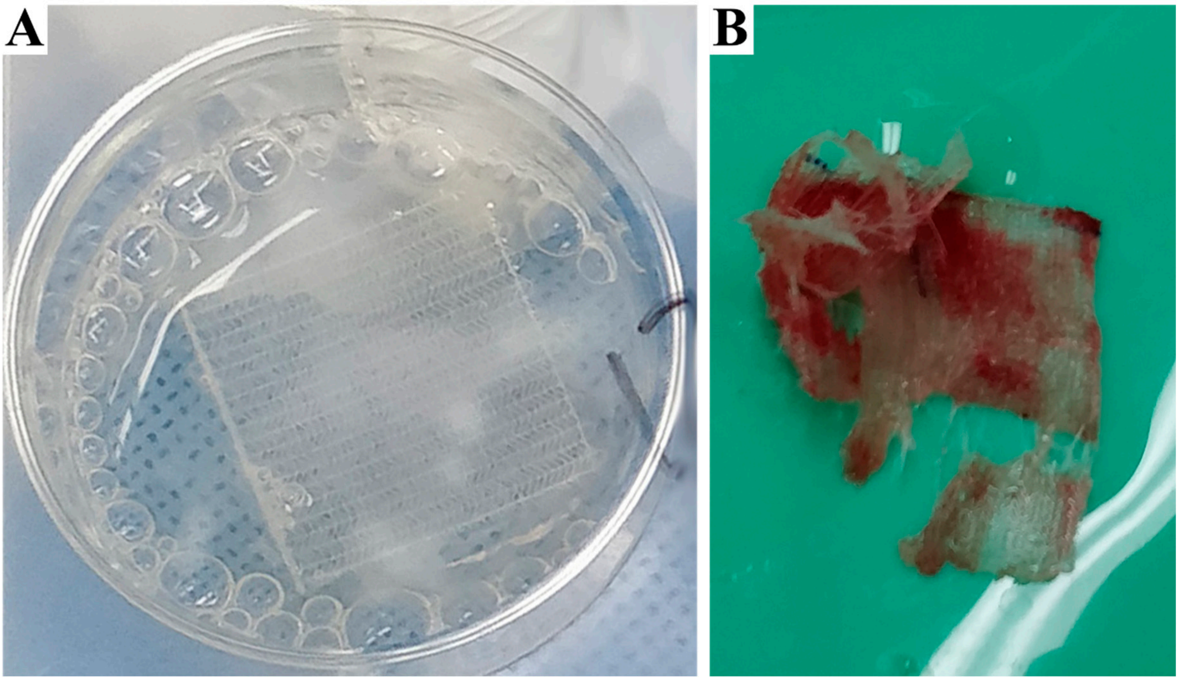

Figure S2. A) Image of mPCL mesh scaffold before implantation. B) image of mPCL mesh scaffold with inferior mechanical properties which were replaced immediately during the implantation surgeries. If the post treatment for surface hydrolyzation and/or sterilization is not performed according to protocol the mechanical properties of the scaffold (A) are reduced significantly. As shown in image B, the manufactured mesh failed already during suturing at the time of surgery. Hence, the failed mesh was immediately retrieved from surgical site and replaced with a new mesh scaffold.

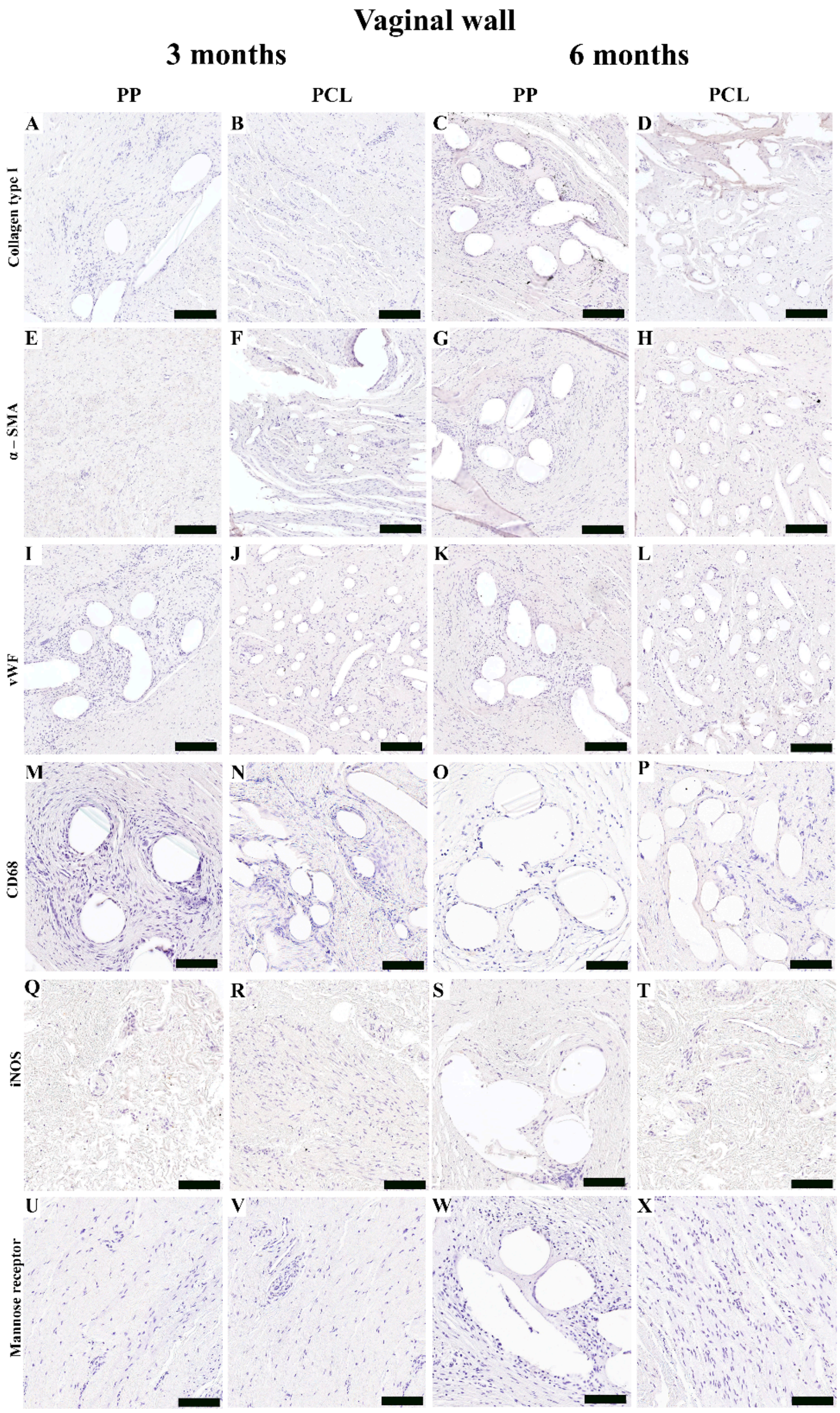

Figure S3. Immunohistochemistry overview of the vaginal wall implanted with the PP mesh and mPCL mesh scaffold at 3- and 6-months' time point isotypes. The cellular responses of the new tissue formed was evaluated using Collagen type I (A – D); anti-smooth muscle actin (α-SMA) (E – H); von Willebrand factor (vWF) (I – L); CD68 macrophages (M – P); inducible nitric oxide synthase (iNOS) (M1) (Q – T); and Mannose receptor (M2) (U – X). The same isotype tissue section was used for COL I vaginal POP and PCL groups 3- months' time point; α-SMA vaginal POP and PCL groups at 3 months' time point; iNOS vaginal POP and PCL groups at 3 months' time point and PCL at 6 months' time point; and Mannose receptor POP and PCL groups 3- months' time point Scale bars: A – L) 200 μm; M – X) 100μm

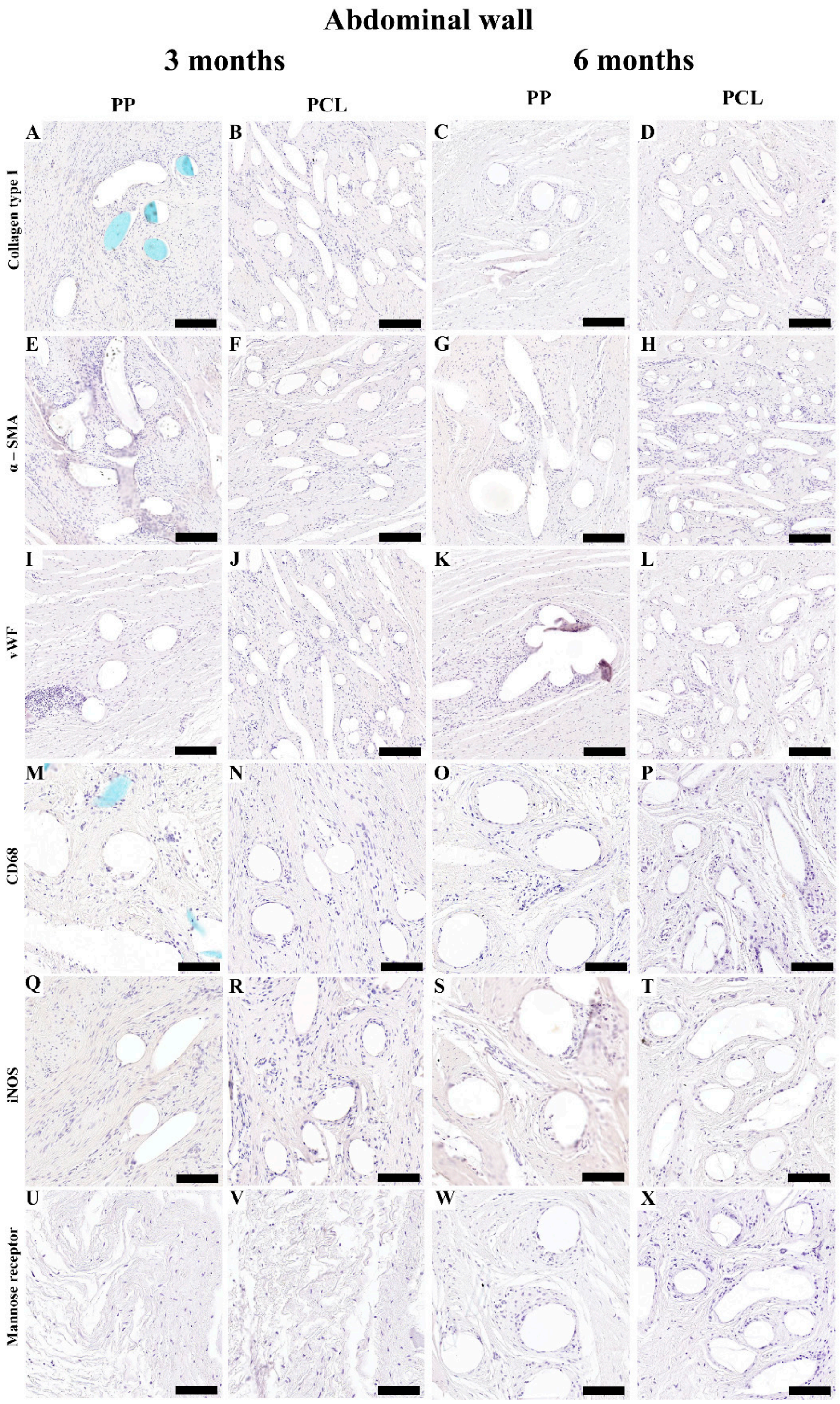

Figure S4. Immunohistochemistry overview of the abdominal wall implanted with the PP mesh and mPCL mesh scaffold at 3- and 6-months' time point isotypes. The cellular responses of the new tissue formed was evaluated using Collagen type I (A – D); anti-smooth muscle actin (α-SMA) (E – H); von Willebrand factor (vWF) (I – L); CD68 macrophages (M – P); inducible nitric oxide synthase (iNOS) (M1) (Q – T); and Mannose receptor (M2) (U – X). Control tissue was used as isotype for Mannose receptor abdominal POP and PCL groups 3- months' time point. Scale bars: A – L) 200 µm; M – X) 100µm
